# Microzymes in Nutrition

**Published:** 1886-06

**Authors:** 


### MICROZYMES IN NUTRITION.

M. BÉCHAMP sums up his views on the action of microzymes in nutrition, and on the origin of ferments in general,—in connection with the prolonged discussion on the ptomaïnes, leucomaïnes, and microbes, and their role in pathology,—in the following clauses:

1. The interior of living bodies is not a passive something, more or less comparable to a vase full of fermentable material. There are not, in the first instance, morbid germs in the air, water, or earth.

2. The organism, living, is so in all its parts, not owing to any occult qualities called *virtue of transformation*, *vital force*, or any other name; but, inasmuch as it is formed of anatomical elements, living *per se*, and, as such, physiologically imperishable and essentially active—which are the microzymes.

3. The organism does not contain foreign attenuated germs of microbes, latent or manifest, unless accidentally introduced; but the microzymes of its different regions and organs become, in certain cases, what are improperly called by the name of microbes.

4. The living body is not hostile to the introduction of micro-organisms, either from outside or from any other new cause; but, thanks to microzymes, it realises the *ensemble of functions that resist death*, of which Bichat spoke.

5. The microzymes, *ab ovo*, explain the chemical and histological phenomena of the organism during its development, produced, not by evolution, but by epigenesis.

6. Microzymes in successively-forming cells, tissues, and organs, change functions at the same time as the cells of those organs. Different again are the microzymes of the blood, the liver, the pancreas, the parotid, the gastric glands, the nervous system, the bones, &c.

7. During these changes the microzymes remain morphologically alike; but their composition changes with their functions. On a line with their development by epigenesis, there is also functional evolution of microzymes in the cells formed by them in the tissues and in the organs.

8. Functional evolution, with a change in the composition of the microzymes, explains why different sorts of cells in the organs, and tissues of the organism, are not nourished in the same way; and why, with the same nutritive materials, they secrete different products, not only in the same being by different organs, but by the same organ in distinct species.

9. The microzymes of a part taken from a living organism may, by evolution, become vibrios, whether in a region artificially cultivated, or in the part itself; and that fact alone ruins entirely all the microbian system.

10. The organism physiologically healthy is that of which the microzymes of all the cells, tissues, and organs, are conformed to an ideal type, not having undergone change by any extra-physiological influence whatever.

11. The microzymes in a given region of an organism during life may undergo vibrional evolution, without for that reason endangering existence in what is essential to it.

12. In tissues, pustules, humours, phlegmos, cysts, &c., when microzymes swarm, whether free or evolved, they proceed from the breaking down of the cells, such breaking down being caused by them when nutrition has been interrupted at its seat.

13. The microzymes becoming, by functional evolution and by relative composition, what they are in each organic centre, may also under various influences—physical, chemical, or moral, through excesses, or privations, or heredity,—undergo in their functions a new development which produces a different temperament, predisposition, or susceptibility, even a different diathesis.

14. The microzymes may also become morbid; that is to say, manifest another order of activity, from which results a disturbance of nutrition which produces a change of focus, the destruction, more or less complete and rapid, of the cells, and the progressive bacterian evolution which follows. This morbid condition may also produce an abnormal cellular proliferation.

15. The morbid microzymes of a given morbid state belong to such and such order of tissues, or organs, without causing those of a different order to become so also.

16. Morbid microzymes of contagious, infectious, or virulent complaints may transmit their state in different ways to microzymes of the same order in a healthy organism,—especially when the predisposition above defined is acquired,—and communicate thus the same disease as that which the case they proceeded from possessed: a disease of which the gravity depends, above all, on the actual state of the subject attacked.

17. Morbid microzymes, whether become bacterian or not, may be cultivated quite as well as healthy microzymes.

18. Morbid microzymes of animals may not transmit their morbidity to microzymes of the same order in an individual of another race of the same sort and the same age, because these have not the same receptivity; but they may communicate the same disease to younger individuals of the same race.

19. The microzymes of two animal species more or less distant are not necessarily functionally identical, either generally or in the same organic centres: that is why morbid microzymes may communicate the disease to one species, and not to another.

20. By regression, or otherwise, the different forms of bacterian evolution of microzymes may return to their initial form of microzymes slightly modified; but then, as in the cultures so in the organism, the acquired morbific function may disappear.

21. After death, when the phenomena of decomposition supervene, *à fortiori*, after total destruction, morbid microzymes, evolved or not, from whatever disease, lose that acquired morbific function, and become of the order of atmospheric microzymes, or those of water, or earth; that is to say, inoffensive.

22. Morbific microzymes, or bacteria, also lose their morbidity by certain cultures, or by applying a suitable temperature. This morbidity they lose little by little, and that is what is called the attenuation of the microbe.

23. It follows from these two last facts that only accidentally can morbific microzymes exist in the air: they cannot normally exist in it.

24. The microzymes of the alimentary canal essentially represent microzymes of digested food, and those that proceed from our own tissues and cells of the intestinal epitehlium. The microzymes of the buccal cavity, and the bacteria found there, also come essentially from the cells of that cavity, &c.

25. Morbid microzymes, evolved or not, may again become healthy; that is to say, by an inverse change of function they may progressively return to their normal mode of nutrition. In this consist cure and return to health.

26. It is necessary to distinguish diseases really parasitic from microzyme diseases; these latter are not parasitic, otherwise we should have to say that parasites make us living.

27. Antiseptics are useful, not to prevent the toxic action of pretended atmospheric microbes, but to prevent or efface the morbid functional evolution of microzymes proper, placed by traumatism or other circumstances in an abnormal situation.

M. Béchamp appears to be entirely opposed to the microbian doctrines of M. Pasteur, the theory of microzymes being exactly opposed to that of the microbes. The latter supposes that the pathogenic microbes pre-exist in the air, at least in the form of germs; whereas the former supposes that the air does not contain them at first, but that our organs contain the microzymes, the germs which are capable of developing vibrios by evolution.—*Bulletin de l'Académie de Médecine*, No. 16, April 20th, 1886.

